# Long-term outcomes of survivors with influenza A H1N1 virus-induced severe pneumonia and ARDS: a single-center prospective cohort study

**DOI:** 10.3389/fcimb.2024.1378379

**Published:** 2024-03-28

**Authors:** Xiao Tang, Xiao-Li Xu, Na Wan, Yu Zhao, Rui Wang, Xu-Yan Li, Ying Li, Li Wang, Hai-Chao Li, Yue Gu, Chun-Yan Zhang, Qi Yang, Zhao-Hui Tong, Bing Sun

**Affiliations:** ^1^ Department of Respiratory and Critical Care Medicine, Beijing Institute of Respiratory Medicine and Beijing Chao-Yang Hospital, Capital Medical University, Beijing, China; ^2^ Department of Radiology, Beijing Chao-Yang Hospital, Capital Medical University, Beijing, China

**Keywords:** influenza A (H1N1) virus, severe community-acquired pneumonia (SCAP), acute respiratory distress syndrome (ARDS), pulmonary fibrosis, pulmonary function

## Abstract

**Introduction:**

Systematic evaluation of long-term outcomes in survivors of H1N1 is still lacking. This study aimed to characterize long-term outcomes of severe H1N1-induced pneumonia and acute respiratory distress syndrome (ARDS).

**Method:**

This was a single-center, prospective, cohort study. Survivors were followed up for four times after discharge from intensive care unit (ICU) by lung high-resolution computed tomography (HRCT), pulmonary function assessment, 6-minute walk test (6MWT), and SF-36 instrument.

**Result:**

A total of 60 survivors of H1N1-induced pneumonia and ARDS were followed up for four times. The carbon monoxide at single breath (D_LCO_) of predicted values and the 6MWT results didn’t continue improving after 3 months. Health-related quality of life didn’t change during the 12 months after ICU discharge. Reticulation or interlobular septal thickening on HRCT did not begin to improve significantly until the 12-month follow-up. The D_LCO_ of predicted values showed negative correlation with the severity degree of primary disease and reticulation or interlobular septal thickening, and a positive correlation with physical functioning. The D_LCO_ of predicted values and reticulation or interlobular septal thickening both correlated with the highest tidal volume during mechanical ventilation. Levels of fibrogenic cytokines had a positive correlation with reticulation or interlobular septal thickening.

**Conclusion:**

The improvements in pulmonary function and exercise capacity, imaging, and health-related quality of life had different time phase and impact on each other during 12 months of follow-up. Long-term outcomes of pulmonary fibrosis might be related to the lung injury and excessive lung fibroproliferation at the early stage during ICU admission.

## Introduction

Influenza has threatened human health for decades (Taubenberger and Morens, 2006). It is estimated that global seasonal influenza-associated respiratory deaths account for 4.0–8.8 per 100,000 individuals annually (Iuliano et al., 2018). During the influenza A (H1N1) virus pandemic in the United States in 2009, 26% of patients with H1N1 pneumonia progressed to acute respiratory distress syndrome (ARDS), and 52% of them were admitted to intensive care unit (ICU) (Jain et al., 2012). The study from Mexico and Canada during the 2013–2014 influenza season showed that the 90-day mortality of critical illness in ICUs was 34.6% (Dominguez-Cherit et al., 2016). It is important to understand the long-term outcomes in survivors.

A study from Canada that focused on the one-year outcomes in survivors of ARDS showed that carbon monoxide diffusion capacity remained low throughout the 12-month follow-up (Herridge et al., 2003). At the 5-year follow-up, pulmonary function was normal to near-normal, but none of the survivors returned to normal predicted levels of physical function (Herridge et al., 2011). Another study from Spain on survivors of ARDS with 6-month follow-up showed a poorer health-related quality of life and mild to moderate pulmonary functional abnormalities compared with the healthy population (Masclans et al., 2011). Similar results were found in survivors among H1N1 patients during the 12-month follow-up (Luyt et al., 2012). However, most studies focused on long-term outcomes in the survivors of H1N1 during the 2009 pandemic. In addition, there are still fewer systematic evaluations of the physical function, pulmonary imaging, and quality of life in such patients. There have been many cases of critical illness of severe H1N1 pneumonia and ARDS in every flu season after 2009 (Iuliano et al., 2018; Li et al., 2019).

This study aimed to characterize long-term outcomes in survivors of severe H1N1 pneumonia and ARDS during 12 months of follow-up. And try to explore the relationship between the long-term pulmonary function and exercise capacity, health-related quality of life, pulmonary imaging, with clinical situation and serological biomarkers acquired in the early stage while ICU admission.

## Methods

### Study design and patients

This was a single-center, prospective, cohort study conducted in a 16-bed respiratory ICU. Patients admitted to the ICU from March 1, 2016, to December 31, 2020, were included in the study. Patients were included if they fulfilled the following criteria: (1) age above 18 years; (2) severe community acquired pneumonia (SCAP) (Cao et al., 2018); (3) meeting the Berlin definition of ARDS (Ferguson et al., 2012); and (4) detection of influenza A (H1N1) virus in sputum or bronchoalveolar lavage fluid (BALF) using real-time polymerase chain reaction (PCR). Exclusion criteria included expected ICU duration less than 48 hours and refuse to participate this study or follow-up.

This study was reviewed and approved by the Ethics Committee of Beijing Chao-Yang Hospital (2016-KE-61). Informed consent was obtained from the patients themselves or their legal guardians. All methods were carried out in accordance with relevant guidelines and regulations.

### Procedures and data collection

Demographic and clinical data of the patients during ICU stay were entered into an electronic case report form (eCRF) and included the following: demographic characteristics, underlying diseases, comorbidities, clinical symptoms, vital signs, laboratory tests, images of the lung, and microbiological findings. Antimicrobial therapy, respiratory support, complications, and outcomes were also recorded. The acute physiology and chronic health evaluation (APACHE) II, sequential organ failure assessment (SOFA), and acute lung injury score (Murray et al., 1988) was also collected while ICU admission.

The survivors were examined during four follow-up visits at outpatient department during 12 months, specifically at 1 month, at 3 months, at 6 months, and at 12 months after ICU discharge. Symptoms and vital signs were recorded. The patients underwent blood routine test, arterial blood gas (ABG) analysis, pulmonary high-resolution computed tomography (HRCT), pulmonary function assessment, and 6-minute walk test (6MWT) at every follow-up visit. Moreover, a 36-item short-form health survey (SF-36) (Skinner et al., 2015) was used to evaluate the physical and mental health function. All of the results were recorded in the eCRF. The diagnose of pulmonary function was referred to Chinese experts’ consensus on the standardization of adult lung function diagnosis (Lei and Rong-Chang, 2022) and ERS/ATS technical standard on interpretive strategies for routine lung function tests (Stanojevic et al., 2022). 6MWT performed according to Chinese expert consensus on standardized clinical application of 6-minute walk test (Chinese Society of Cardiology CMA et al., 2022).

The primary outcome was the incidence of patients with abnormal pulmonary function at 12-month follow-up. The secondary outcome was the symptom, HRCT manifestation, pulmonary function, SF-36, and 6MWT at 4 times follow-up.

### Pulmonary HRCT

We performed lung segmentation, lesion extraction and labeling, and volume calculation using a dedicated multi-task deep learning algorithm developed for pulmonary pneumonia (Beijing Deepwise & League of PhD Technology Co.Ltd, China). One experienced radiologist with experience in pulmonary imaging interpretation reviewed the CT images. The CT images were evaluated and defined according to the Fleischner Society glossary of terms for thoracic imaging (Hansell et al., 2008) as the following radiologic patterns: (a) ground-glass opacification (GGO); (b) consolidation; (c) bronchiectasis; (d) reticulation or interlobular septal thickening; and (e) emphysema. The extent of disease at HRCT was evaluated as a CT score. Bilateral lungs were divided into five lung zones, where each lung zone was assigned a score that was based on the following: score 0, 0% involvement; score 1, less than 25% involvement; score 2, 25% to less than 50% involvement; score 3, 50% to less than 75% involvement; and score 4, 75% or greater involvement. Summation of scores provided overall lung involvement (maximal CT score for both lungs was 20) (Ooi et al., 2004).

### Measurement of inflammatory and fibrogenic cytokines

Serum specimens were collected dynamically during the first nine days for the measurement of inflammatory cytokines and fibrogenic cytokines. A human cytokine panel (Procarta Plex™, Affmetrix Inc., CA, USA) consisting of IFN-γ, IL-12p70, IL-13, IL-1b, IL-2, IL-4, IL-5, IL-6, TNF-α, GM-CSF, IL-18, IL-10, IL-17A, IL-21, IL-22, IL-23, IL-27, IL-9, IFN-α, IL-15, IL-1a, IL-1RA, IL-7, TNF-β, and IL-31 was used to measure inflammatory cytokines. Fibrogenic cytokines consisted of hyaluronic acid, laminin, type IV collagen, type III procollagen (Bioscience, Tianjin, China), and Krebs Von den Lungen-6 (KL-6) (Fujirebio Inc., Tokyo, Japan).

### Statistical analysis

Data analysis was performed using SPSS 23.0 (IBM Corp., Armonk, NY, USA) software. Categorical variables were summarized using frequencies and percentages, and continuous data were presented as the medians (interquartile ranges [IQRs]). The Mann–Whitney *U* test was used for continuous variables, and the χ2 test or Fisher’s exact test was used for categorical variables. Differences between groups were tested by one-way analysis of variance test. The overall time course for pulmonary function, HRCT manifestations, and eight dimensions of the SF-36 instrument was analyzed using two-way analysis of variance for repeated measures. Pearson correlation analysis was used to analyze the correlation between cytokine levels, pulmonary function, pulmonary manifestations on HRCT, and dimensions of the SF-36 instrument in four visits during the 12-month follow-up after ICU discharge. Stepwise regression and collinearity diagnostics were used to analyze the multicollinearity among the variables. *P* values lower than 0.05 were considered to be statistically significant.

## Results

From March 1, 2016, to December 31, 2019, a total of 345 patients with SCAP and ARDS were admitted to the respiratory ICU of Beijing Chao-Yang Hospital. In total, 92 (26.7%) of them were diagnosed with influenza A (H1N1) virus–induced SCAP, of which 70 (76.1%) patients were male, and the median age was 49 (41, 63) years. The median acute lung injury score was 3.25 (2.75, 3.74), and Acute Physiology and Chronic Health Evaluation (APACHE) II was 12 (9, 18) at the time of admission. The median PaO_2_/FiO_2_ was 107.5 (77.0, 137.8) mm Hg. All of the patients received mechanical ventilation during ICU therapy. Invasive mechanical ventilation (IMV) was used in 70 (76.1%) patients, including 38 (41.3%) patients who received extracorporeal membrane oxygenation (ECMO) support. The ICU mortality was 34.8%, and the median length of ICU stay was 16 (10, 27) days (**Supplementary Table S1**). Sixty survivors underwent four follow-up examinations at 1, 3, 6, and 12 months after ICU discharge. Six patients were lost to follow-up, and one patient died for cerebrovascular disease (**Supplementary Figure S1**).

### Symptoms, pulmonary function, and 6MWT

At the one-month follow-up, 39.3% of the survivors had cough. The number of patients with respiratory tract symptoms decreased gradually with time. Fourteen survivors (24.0%) had hypoxemia at the one-month follow-up, which decreased to four patients (7.0%) at the three-month follow-up. However, at the 12-month follow-up, there were still four patients with cough, and one patient had dyspnea and hypoxemia (**Table 1**).

**Table 1:** Symptoms, pulmonary function, and 6MWT during 12 months of follow-up.

Follow-up	1 month (n = 58)	3 months (n = 57)	6 months (n = 54)	12 months (n = 53)	*P*
Symptom (n, %)					
Cough	24 (39.3)	12 (21.0)	7 (12.7)	4 (7.5)	0.416
Sputum	15 (24.6)	8 (14.0)	6 (10.9)	3 (5.7)	0.969
Short breath	4 (6.6)	3 (5.3)	0	1 (1.9)	0.537
Dyspnea	8 (13.1)	0	0	1 (1.9)	0.094
Arterial blood gas analysis					
pH	7.41 (7.39, 7.43)	7.40 (7.39, 7.42)	7.41 (7.40, 7.41)	7.41 (7.39, 7.42)	0.820
PaCO_2_ (mm Hg)	32.0 (31.2, 34.5)	32.4 (30.7, 33.5)	33.8 (30.6, 34.9)	35.8 (33.7, 37.2)	0.660
PaO_2_ (mm Hg)	89.2 (74.4, 95.9)	91.9 (86.8, 99.2)	88.1 (79.3, 93.5)	89.7 (76.5, 93.4)	0.489
PaO_2_/FiO_2_ (mm Hg)	406.8 (354.1, 455.2)	439.7 (410.5, 474.4)	421.5 (379.5, 447.4)	428.2 (365.3, 446.4)	0.431
Hypoxemia (n, %)	14 (24.0)	4 (7.0)	3 (5.4)	1 (1.9)	0.001
Pulmonary function					
FVC (L)	3.30 (2.51, 3.84)	3.74 (2.87, 4.26) *	3.87 (3.09, 4.22) *	3.82 (3.17, 4.26)	0.009
Difference		0.560 (0.143, 0.977)	0.185 (0.005, 0.364)	0.075 (−0.151, 0.302)	
FVC% of predicted value	81.8 (71.6, 89.4)	97.2 (81.7, 101.3) *	97.2 (87.6, 103.0) *	93.9 (89.0, 112.4)	0.011
Difference		17.445 (4.533, 30.358)	5.327 (0.356, 10.299)	3.173 (−3.277, 9.622)	
FEV 1 (L)	2.72 (2.15, 3.18)	3.02 (2.42, 3.50) *	2.96 (2.56, 3.45)	3.00 (2.54, 3.28)	0.038
Difference		0.385 (0.058, 0.711)	0.106 (−0.039, 0.252)	0.045 (−0.109, 0.198)	
FEV 1% of predicted value	82.0 (73.1, 90.4)	93.1 (82.2, 101.2) *	97.2 (83.9, 101.4) *	92.9 (83.0. 108.8)	0.024
Difference		14.300 (0.570, 28.030)	5.845 (1.100, 10.590)	1.464 (−4.870, 7.797)	
FEV 1/FVC	85.8 (81.2, 89.0)	84.5 (80.9, 96.2)	85.7 (82.4, 97.5)	85.0 (79.9, 95.3)	0.558
Difference		1.609 (−7.952, 1.170)	−1.736 (−15.754, 12.282)	−1.855 (−7.188, 3.479)	
MEF75/25	2.90 (2.15, 4.00)	3.33 (2.22, 3.90)	2.76 (2.09, 3.27)	2.48 (2.06, 3.75)	0.787
Difference		0.098 (−0.777, 0.974)	0.020 (−0.620, 0.660)	−0.027 (−0.364, 0.311)	
MEF 75/25% of predicted value	75.95 (57.1, 96.9)	79.1 (62.5, 100.3)	73.5 (61.2, 99.0)	72.1 (55.3, 100.9)	0.788
Difference		4.063 (−24.678, 32.804)	1.427 (−18.643, 21.516)	−0.850 (−11.580, 9.880)	
TLC (L)	4.51 (3.53, 5.30)	5.23 (3.88, 5.79)	5.16 (4.39, 6.08)	5.20 (4.25, 5.90)	0.033
Difference		1.267 (−0.098, 2.633)	0.175 (−0.401, 0.752)	0.088 (−0.172, 0.349)	
TLC% of predicted value	73.65 (63.85, 86.15)	84.1 (76.1, 92.7) *	90.3 (76.9, 95.1)	92.3 (84.6, 99.4)	<0.001
Difference		14.320 (4.233, 24.407)	5.440 (−0.210, 11.090)	1.380 (−3.833, 6.593)	
D_LCO_ (mL/min/mm Hg)	5.29 (3.42, 6.99)	6.15 (5.00, 7.90)	6.44 (5.74, 7.86)	6.78 (6.06, 8.52)	<0.001
Difference		1.022 (−1.027, 3.070)	0.994 (−0.511, 2.499)	0.355 (−0.334, 1.043)	
D_LCO_% of predicted value	56.7 (45.7, 69.0)	71.9 (59.0, 82.9) *	73.7 (68.3, 86.8)	81.2 (73.8, 90.8)	<0.001
Difference		23.364 (8.953, 37.774)	5.900 (−4.602, 16.402)	3.882 (−5.510, 13.273)	
D_LCO_/VA (mL/min/mm Hg/L)	1.33 (1.13, 1.54)	1.28 (1.11, 1.56)	1.37 (1.22, 1.62)	1.60 (1.22, 1.79)	0.584
Difference		−0.189 (−1.349, 0.971)	0.076 (−0.144, 0.296)	11.326 (−26.684, 49.336)	
D_LCO_/VA% of predicted value	77.5 (67.1, 91.9)	84.3 (75.2, 94.6) *	88.3 (79.0, 104.9)	95.2 (80.5, 111.7)	0.001
Difference		16.3 (5.2, 27.5)	5.2 (−4.5, 14.9)	2.9 (−5.2, 11.1)	
6MWT	450 (372, 519)	540 (421, 597) *	534 (493, 600) *	538 (430, 604)	0.005
		135 (56, 214)	76 (98, 250)	−5 (70, 61)	

P: Two-way analysis of variance for repeated measures to analyze the difference between four visits during 12 months.

One-way analysis of variance was used to analyze differences between subgroups. Differences showed the comparison with the last follow-up visit. *: with significant difference compared with the result of the last follow-up visit.

D_LCO_, diffusion capacity of the lung for carbon monoxide at single breath; D_LCO_/VA, diffusion capacity of the lung for carbon monoxide corrected by alveolar volume; FiO_2_, fraction of inspired oxygen; FEV 1, the first-second forced expiratory volume; FVC, forced vital capacity; MEF 25, maximum expiratory flow rate at 25% vital capacity; MEF 50, maximum expiratory flow rate at 50% vital capacity; MEF 75, maximum expiratory flow rate at 75% vital capacity; 6MWT, 6-minute walk test; PaCO_2_, arterial partial pressure of carbon dioxide; PaO_2_, arterial partial pressure of oxygen; and TLC, total lung capacity.

**Figure 1:**
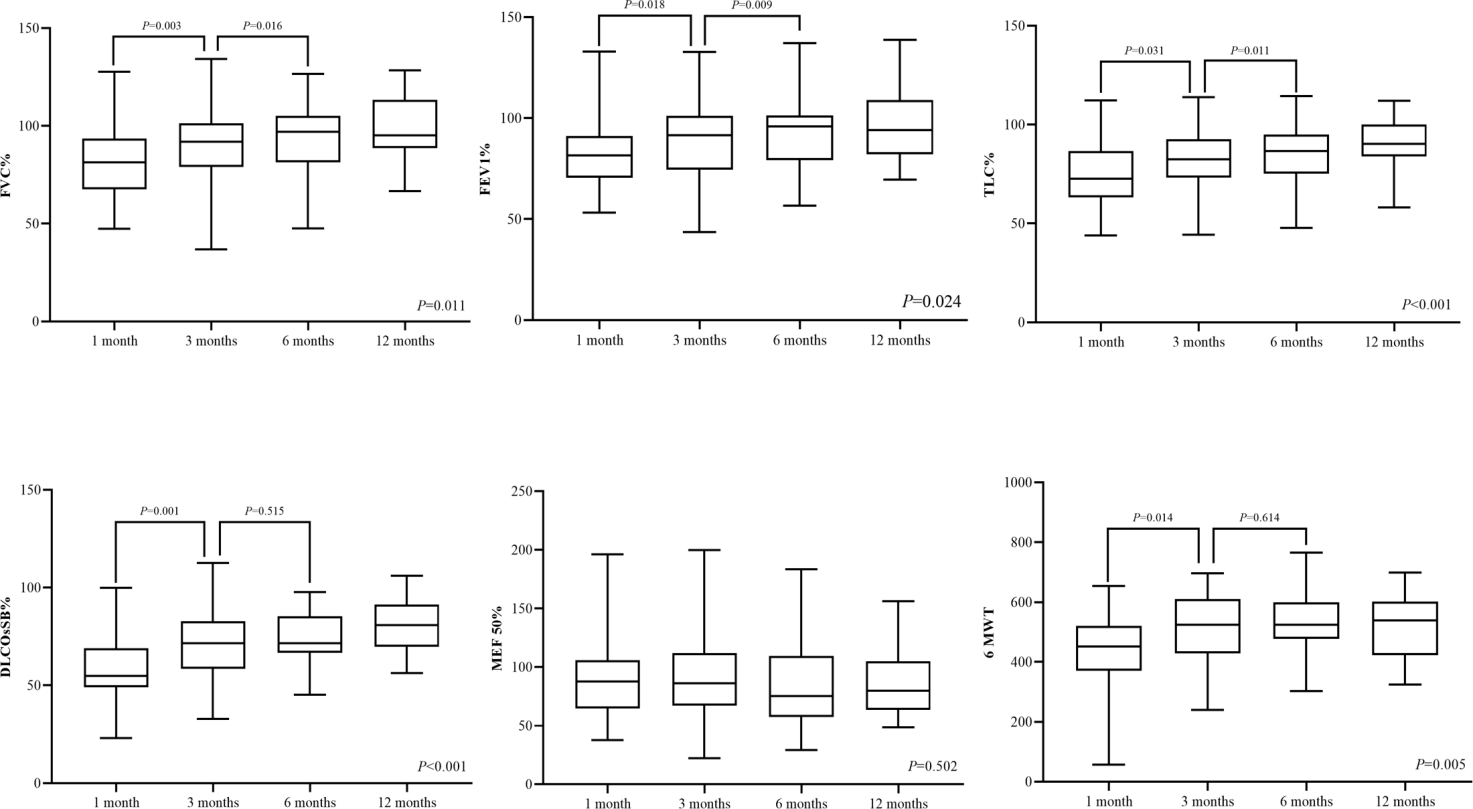
Pulmonary function assessment and 6MWT in four visits during 12 months of follow-up. D_LCO_, diffusion capacity of the lung for carbon monoxide at single breath; TLC, total lung capacity; FEV 1, the first-second forced expiratory volume; FVC, forced vital capacity; MEF 50, maximum expiratory flow rate at 50% vital capacity; and 6MWT, 6-minute walk test.

Pulmonary function assessment at the one-month follow-up revealed gas transfer impairments in 32 patients (55.2%), of which 24 patients (41.4%) had limited ventilation, while eight patients had small-airway dysfunction. The dynamic evaluation showed that the pulmonary function significantly improved at the 3-month follow-up compared with the 1-month follow-up. Single breath diffusing capacity of lung for carbon monoxide (D_LCO_) of predict values was 71.9% (59.0%, 82.9%) at the 3-month follow-up, significantly higher than 56.7% (45.7%, 69.0%) at the 1-month follow-up. However, the comparison between 3-month, 6-month, and 12-month follow-up showed no significant difference. At the 12-month follow-up, there were still 12 survivors (21.8%) with gas transfer impairments, in whom D_LCO_ of predict values was 69.7% (66.1%, 75.2%). Seven (12.7%) patients had small-airway dysfunction, and four (7.3%) patients had limited ventilation. The distance at 6MWT at the 1-month follow-up was 450 m (372 m, 519 m), which increased significantly to 540 m (421 m, 597 m) at the 3-month follow-up but did not further improve at the 6-month and 12-month follow-up (**Table 1** and **Figure 1**).

### Physical and mental health function

**Figure 2:**
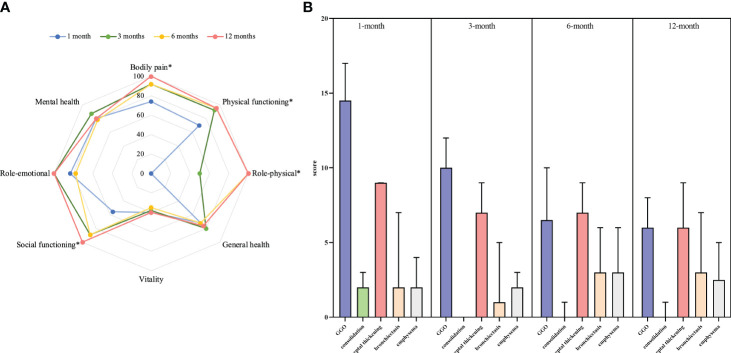
SF-36 and pulmonary HRCT scores in four visits during 12 months of follow-up of the survivors of H1N1 virus–induced SCAP and ARDS. **(A)** Eight dimensions of the SF-36 instrument. *: Two-way analysis of variance for repeated measures to analyze the difference between the four visits showed significant within-group differences. **(B)** Pulmonary HRCT scores of different manifestations. GGO, ground-glass opacity; HRCT, high-resolution computed tomography.

The SF-36 instrument was used to evaluate the physical and mental health function. At the 1-month follow-up, the SF-36 instrument showed poor manifestations in eight dimensions of physical and mental health function. Physical functioning and social functioning significantly improved at the 3-month follow-up. Then, at 6 months after ICU discharge, bodily pain and role–physical significantly improved. However, general health, vitality, mental health, and role–emotion did not change during 12 months after ICU discharge (**Table 2** and **Figure 2A**).

**Table 2:** The scores in the SF-36 instrument during 12 months of follow-up.

Follow-up	1 month (n = 58)	3 months (n = 57)	6 months (n = 54)	12 months (n = 53)	*P*
Bodily pain	74 (70, 100)	92 (74, 100)	92 (84, 100) *	100 (82, 100)*	0.030
Difference		16 (−1, 32)	4 (−4, 12)	−1 (−8, 5)	
Physical functioning	70 (55, 90)	92 (80, 95) *	95 (85, 95) *	95 (82, 98) *	0.050
Difference		23 (5, 41)	3 (−2, 8)	2 (−7, 12)	
Role–physical	0 (0, 0)	50 (0, 100)	100 (25, 100)*	100 (75, 100)*	0.002
Difference		32 (−12, 77)	15 (−13, 43)	25 (−4, 54)	
General health	72 (50, 82)	80 (57, 97)	72 (52, 87)	76 (57, 95)	0.118
Difference		5 (−6, 16)	13 (−1, 27)	2 (−13, 17)	
Vitality	40 (25, 55)	38 (20, 50)	35 (25, 40)	40 (35, 50)	0.262
Difference		6 (−12, 23)	−14 (−30, 2)	3 (−8, 14)	
Social functioning	55.6 (33.3, 77.8)	88.9 (66.7, 100) *	89 (78, 100) *	100 (89, 100) *	0.004
Difference		21 (4, 38)	9 (−8, 26)	2 (−9, 13)	
Role–emotional	83.3 (0, 100)	100 (66.7, 100)	78 (68, 88)	100 (83, 100)	0.258
Difference		23 (−10, 57)	17 (−6, 39)	−14 (−37, 9)	
Mental health	80 (64, 88)	87 (72, 92)	78 (68, 88)	80 (68, 92)	0.382
Difference		4 (−10, 17)	−1 (−7, 5)	−8 (−24, 8)	
Reported health transition	25 (0, 25)	37.5 (25, 75)	50 (25, 100)*	75 (25, 100)*	0.003
Difference		22 (−2, 47)	27 (−6, 60)	−3 (−32, 27)	

P: Two-way analysis of variance for repeated measures to analyze the difference between four visits during 12 months.

One-way analysis of variance was used to analyze differences between subgroups. Differences showed the comparison with the last follow-up visit. *: there was significant difference of the result compared with the 1-month follow-up.

### Pulmonary HRCT

The median patient’s whole-lung volume by volumetric HRCT analysis was 3352 (2241, 3893) ml at the 1-month follow-up, which increased to 3769 (2696, 4643) ml at the 3-month follow-up (*P* = 0.017). At the one-month visit, the main pulmonary HRCT manifestation was GGO, of which the median score was 14 (10, 18). GGO improved significantly at the next three follow-up visits (*P* < 0.001). Consolidation was fully disappeared at 3 months after ICU discharge. However, bronchiectasis, reticulation, or interlobular septal thickening did not begin to improve significantly until the 12-month follow-up (**Table 3** and **Figures 2B**, **Supplementary Figure S2**).

**Table 3:** Pulmonary HRCT descriptions during 12 months of follow-up.

HRCT manifestation	1 month (n = 58)	3 months (n = 57)	6 months (n = 54)	12 months (n = 53)	*P*
GGO	14 (10, 18)	10 (6, 16)*	9 (4, 11)*	6 (2, 10)*	<0.001
Difference		−3 (−5, −1)	−3 (−6, −1)	−2 (−5, 1)	
Consolidation	3 (2, 5)	0 (0, 4)*	0 (0, 3)*	0 (0, 2)*	0.015
Difference		−2 (−4, 0)	0 (−1, 0)	0 (−1, 0)	
Reticulation/interlobular septal thickening	9 (6, 11)	9 (6, 10)	8 (6, 10)	6 (5, 9) **	0.031
Difference		0 (−1, 1)	−1 (−2, 0)	−1 (−1, 0)	
Bronchiectasis	6 (2, 11)	5 (2, 9)	5 (1, 8)	4 (1, 8) **	0.018
Difference		−1 (−3, 0)	−1 (−1. 0)	0 (−1, 0)	
Emphysema	4 (1, 8)	4 (2, 8)	4 (2, 9)	4 (2, 10)	0.123
Difference		0 (−1, 1)	0 (−1, 1)	0 (0, 1)	
Lung volume	3352 (2241, 3893)	3769 (2696, 4643)*	3537 (2486, 4840)*	4012 (2872, 4721)*	0.004
Difference		584 (173, 995)	−31 (−287, 225)	174 (−105, 453)	

P: Two-way analysis of variance for repeated measures to analyze the difference between four visits during 12 months.

One-way analysis of variance was used to analyze differences between subgroups. Differences showed the comparison with the last follow-up visit. *: the result of this follow-up with significant difference compared with the 1-month follow-up. **: the result of this follow-up with significant difference compared either with the 1-month follow-up, or 3-month follow-up.

GGO, ground-glass opacity; HRCT, high-resolution computed tomography.

### Correlation analysis

**Figure 3:**
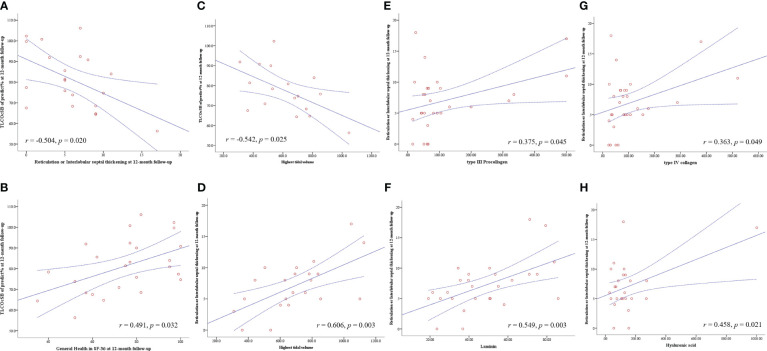
Pearson correlation analysis was used to analyze the correlation between the fibrogenic cytokines and variables during ICU therapy, pulmonary function, pulmonary HRCT, and SF-36 instrument in four visits during 12 months of follow-up after ICU discharge. The solid line is the fitted line of Pearson correlation analysis; the dotted line is the 95% confidence interval. D_LCO_, diffusion capacity of the lung for carbon monoxide at single breath; HRCT, high-resolution computed tomography; and ICU, intensive care unit.

D_LCO_ of predict values at four follow-up examinations showed a moderate negative correlation with reticulation or interlobular septal thickening manifested on HRCT at the corresponding time point (**Figure 2B**). Other indicators of pulmonary function and HRCT manifestations did not correlate. There were moderate to strong positive correlations between D_LCO_ of predict values and physical functioning (*r* = 0.683, *P* < 0.001), role–physical (*r* = 0.622, *P* < 0.001), role–emotional (*r* = 0.507, *P* = 0.003), and 6MWT (*r* = 0.522, *P* = 0.004) at the 3-month follow-up (**Supplementary Table S2**). At the 12-month visit, D_LCO_ of predict values still showed moderate positive correlations with role–physical (*r* = 0.431, *P* = 0.032) and general health (*r* = 0.491, *P* = 0.013) (**Supplementary Table S2** and **Figures 3A, B**).

Regarding the variables during ICU hospitalization, acute lung injury score (*r* = −0.471, *P* = 0.017) and APACHE II during ICU admission (*r* = −0.691, *P* < 0.001) had a negative correlation with D_LCO_ of predict values at the 12-month follow-up. The highest tidal volume was the only monitoring parameter during mechanical ventilation that had a negative correlation with D_LCO_ of predict values (*r* = −0.542, *P* = 0.025) and a positive correlation with reticulation or interlobular septal thickening (*r* = 0.606, *P* = 0.003) at the 12-month follow-up (**Figures 3C, D**). Regarding the fibrogenic cytokines at the early stage of ICU admission, KL-6 level showed moderately positive correlations with reticulation or interlobular septal thickening at 1-month and 3-month follow-up, while type IV collagen, type III procollagen, hyaluronic acid, and laminin had moderate to strong positive correlations with reticulation or interlobular septal thickening at 6 months and 12 months after ICU discharge (**Supplementary Table S3** and **Figures 3E–H**). There were no correlations between inflammatory cytokines and HRCT manifestations. Very weak multicollinearity among the above variables was shown in the collinearity diagnostics (**Supplementary Table S4**).

## Discussion

This study systematically assessed the long-term outcomes of pulmonary function, exercise capacity, HRCT manifestations, and health-related quality of life in survivors of severe H1N1 pneumonia and ARDS who had been discharged from ICU. The improvements in pulmonary function and exercise capacity, HRCT, and health-related quality of life had different time phase and impact on each other. The decrease in D_LCO_ was the main abnormality in pulmonary function, which improved at 3 months after ICU discharge. Although GGO and consolidation were fully absorbed at 3 months after ICU discharge, reticulation or interlobular septal thickening was the main long-term manifestation on HRCT, which did not begin to improve significantly until the 12-month follow-up, and had a negative correlation with D_LCO_. Regarding health-related quality of life, general health and mental health were without any changes during one year after ICU discharge, even though there was a positive correlation between D_LCO_ and general health. The severity of primary disease, tidal volume of mechanical ventilation, and levels of fibrogenic cytokines at the early stage of ICU admission affected the long-term outcomes of reticulation or interlobular septal thickening on HRCT.

A study from Greece evaluated 44 patients with H1N1 infection every three months, until 6 months after discharge. There was an improvement in pulmonary function tests at the second measurement, but there were no changes between the second and third measurements (Zarogoulidis et al., 2011). Huang’s team in Taiwan followed up on nine survivors of ARDS due to severe H1N1 pneumonitis at 1, 3, and 6 months after hospital discharge. Pulmonary function and 6MWT results increased from 1 to 3 months after hospital discharge, but there was no further improvement from 3 to 6 months after discharge (Hsieh et al., 2018). Similar results were also found for ARDS of other etiologies. A 3-month follow-up study in survivors of ARDS secondary to coronavirus disease 2019 (COVID-19) showed that 82% of these patients had an impaired D_LCO_ (Gonzalez et al., 2021). Similar to the previous literature, the present study showed that gas transfer impairment was the main manifestation of the abnormal pulmonary function, and in most survivors, it recovered to normal range at 3 months after ICU discharge. However, there were still 25% of patients in whom there was no improvement during 12 months.

Bilateral, peripheral GGO, and/or bilateral areas of consolidation are the predominant HRCT findings in the acute phase of H1N1-induced pneumonia (Marchiori et al., 2010). Short-term serial HRCT evaluation of patients with H1N1 infection showed that GGO and/or consolidation on initial CT scans tended to resolve fibrosis, which then resolved completely or displayed substantially reduced residual disease (Li et al., 2012). Another study showed that the most common HRCT finding in the convalescent stage in patients with H1N1 pneumonia was fibrosis (Feng et al., 2015). Nevertheless, few studies have focused on the long-term radiographic outcomes in survivors of H1N1 infection.

The present study revealed the dynamic HRCT evolution in survivors of H1N1-induced pneumonia and ARDS during 12 months after ICU discharge. GGO and consolidation were the main manifestations on HRCT at the early stage after ICU discharge, similar to that at the acute phase of H1N1 pneumonia. Especially, consolidation was mostly absorbed at the 3-month visit. However, reticulation or interlobular septal thickening did not begin to improve significantly until the 12-month follow-up. It means that reticulation or interlobular septal thickening is the main long-term HRCT finding in H1N1-induced pneumonia and ARDS. A similar result was found in the study of COVID-19 survivors (Gonzalez et al., 2021; Pan et al., 2022). We also discovered that the severity of reticulation or interlobular septal thickening correlated with diffusion capacity in such patients.

A study from Australia showed that health-related quality of life of survivors of severe H1N1 influenza was comparable to that of healthy population one year after ICU discharge (Skinner et al., 2015). However, researchers from Spain proposed a significant but temporary impact on health-related quality of life in the majority of patients with H1N1 infection (Hollmann et al., 2013). According to a review from Bein’s team, surviving ARDS is associated with a long-term substantial reduction in health-related quality of life (Bein et al., 2018). Neufeld’s team also reported that two-thirds of ARDS survivors had clinically significant fatigue symptoms in the first year of follow-up (Neufeld et al., 2020). In this study, we found that general health, vitality, mental health, and role–emotion in survivors of H1N1-induced pneumonia and ARDS did not change during 12 months after ICU discharge, which might be affected partially by impaired pulmonary function, and the experience in ICU might impact long-term mental health. Therefore, for the improvement in health-related quality of life, both psychological and physical recovery should be focused on.

Type III procollagen is recognized as a marker of ARDS-associated lung fibroproliferation (Forel et al., 2015). KL-6 is a marker of alveolar inflammation in ARDS (Nathani et al., 2008), which is also related to long-term outcomes (Kondo et al., 2011). In the present study, KL-6 was related to reticulation or interlobular septal thickening at the 1-month and 3-month visits, but not at the 12-month follow-up. In contrast, type IV collagen, type III procollagen, hyaluronic acid, and laminin showed correlations with reticulation or interlobular septal thickening at 6 months and 12 months after ICU discharge. We considered that the lung injury and excessive lung fibroproliferation at the early stage during ICU admission might be related to long-term pulmonary fibrosis in patients with H1N1-induced ARDS. It also influences the recovery of pulmonary function and quality of life.

Lung injury and excessive lung fibroproliferation at the early stage during ICU admission might not only be related to the severity of the primary disease (Burnham et al., 2014) but also to mechanical ventilation. Many studies have tried to explore the mechanism of ventilation-induced acute lung injury and fibroproliferation in patients with ARDS, which caused poor outcomes, and high tidal volume was identified as one of the important risk factors (Foda et al., 2001; Li et al., 2008; Cabrera-Benitez et al., 2014). Lung-protective mechanical ventilation with lower tidal volumes has been widely used and could decrease the mortality of patients with ARDS (Acute Respiratory Distress Syndrome N et al., 2000). In the present study, all of the patients received standard lung-protective mechanical ventilation; nevertheless, the relationship between the tidal volume and the long-term outcome was still observed, especially influencing the pulmonary function and fibrosis manifestation on HRCT at the 12-month visit. Therefore, strictly executing the mechanical ventilation strategy with lower tidal volumes might help patients with ARDS in achieving better short-term or long-term outcomes. There is still controversy regarding the effectiveness of corticosteroids applied to reduce ventilation-induced lung fibrosis in severe H1N1 infection–induced ARDS (Burnham et al., 2014; Cabrera-Benitez et al., 2014; Moreno et al., 2018). Thus, effective pharmacological therapies to prevent the development of ventilator-induced and ARDS-related lung fibrosis should be the research priority in the future.

There are still some limitations to this study. First, this was a single-center cohort study, which may have induced the unavoidable selection bias. Second, the sample size was relatively small, which might have resulted in less statistical power. Third, this study focused on patients with H1N1 pneumonia–induced ARDS; therefore, extrapolation of the results to other etiologies of pneumonia and ARDS should be done with caution. Fourth, post-ICU life capability, workability, and delirium which was recognized as aspects related to long-term prognosis after ICU discharge was not collected in the present study. This study only describes the long-term prognosis from the perspectives of imaging, health-related quality of life, and pulmonary function. Lastly, inflammatory cytokines and fibrogenic cytokines were not measured in the bronchoalveolar lavage fluid, so we could only partly explain the injury degree of alveolar epithelial cells through the results from serum.

## Conclusions

The improvements in pulmonary function and exercise capacity, imaging, and health-related quality of life in survivors of critical H1N1-induced pneumonia and ARDS had different time phase and impact on each other during 12 months of follow-up after ICU discharge. Long-term pulmonary fibrosis outcome, which would influence the pulmonary function and quality of life, could be observed via HRCT longitudinal evaluation. It might be not only related to the severity of the primary disease but also to mechanical ventilation. These topics still need to be explored in multicenter studies, covering various etiologies and using larger sample sizes. Moreover, the mechanism also needs to be further investigated.

## Data availability statement

The raw data supporting the conclusions of this article will be made available by the authors, without undue reservation.

## Ethics statement

The studies involving humans were approved by the Ethics Committee of Beijing Chao-Yang Hospital. The studies were conducted in accordance with the local legislation and institutional requirements. The participants provided their written informed consent to participate in this study.

## Author contributions

XT: Conceptualization, Data curation, Investigation, Writing – original draft. X-LX: Conceptualization, Data curation, Investigation, Writing – review & editing. NW: Investigation, Writing – review & editing. YZ: Investigation, Writing – review & editing. RW: Formal analysis, Investigation, Writing – review & editing. X-YL: Investigation, Writing – review & editing. YL: Investigation, Writing – review & editing. LW: Investigation, Writing – review & editing. H-CL: Investigation, Writing – review & editing. YG: Investigation, Writing – review & editing. C-YZ: Investigation, Writing – review & editing. QY: Investigation, Writing – review & editing. Z-HT: Funding acquisition, Writing – review & editing. BS: Conceptualization, Funding acquisition, Writing – review & editing.
