# Supplementary material for: Long-term outcomes of survivors with influenza A H1N1 virus-induced severe pneumonia and ARDS: a single-center prospective cohort study
Source: Front Cell Infect Microbiol. 2024 Mar 28;14:1378379. doi: 10.3389/fcimb.2024.1378379 (PMC11007161; doi:10.3389/fcimb.2024.1378379)
Supplement: Supplementary file 1 [file DataSheet_1.pdf]

Figure S1. Flowchart.

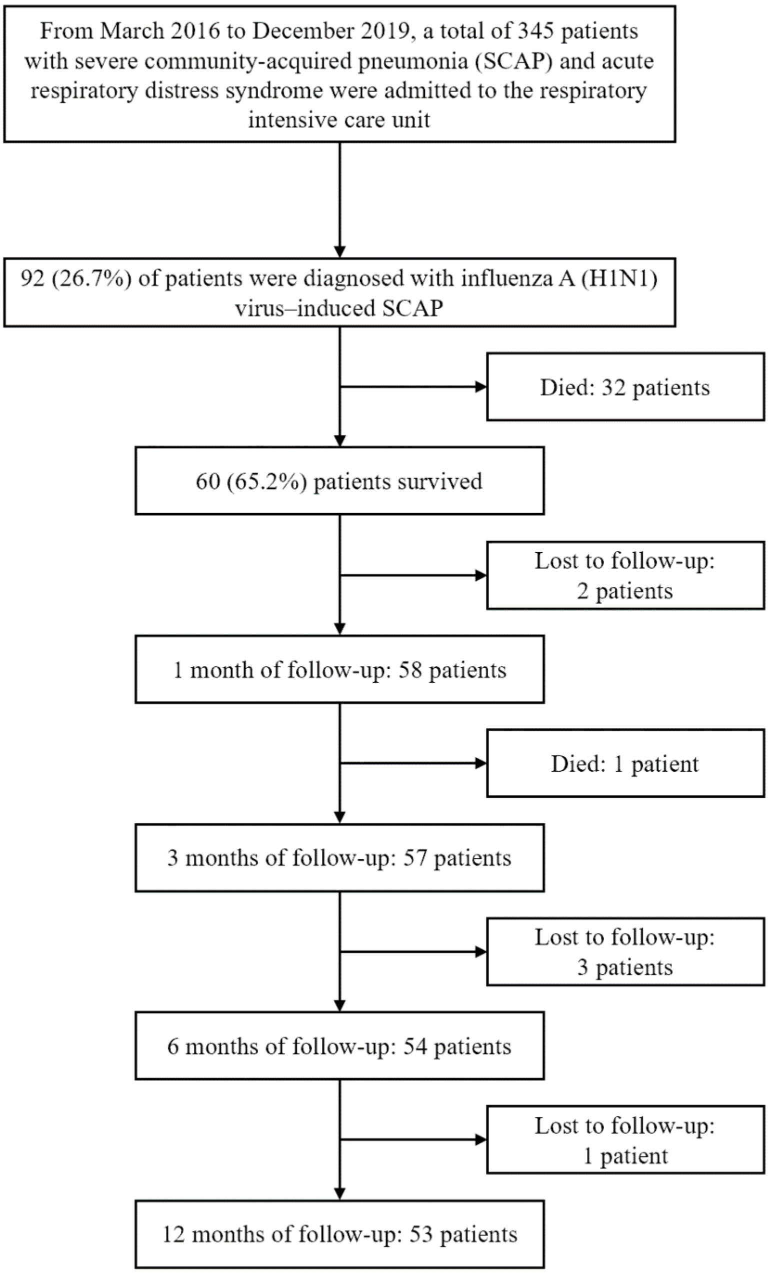

Figure S2. Pulmonary high-resolution computed tomography of survivors who recovered at 3 and 6 months of follow-up and patients unrecovered at 12 months of follow-up.

A. A 34-year-old man whose pulmonary function at the 3-month follow-up was normal.

B. A 38-year-old man whose pulmonary function at the 3-month follow-up showed decreased diffusion capacity of the lung for carbon monoxide but recovered at 6 months after discharge.

C. A 60-year-old man who did not recover by 12 months after discharge.

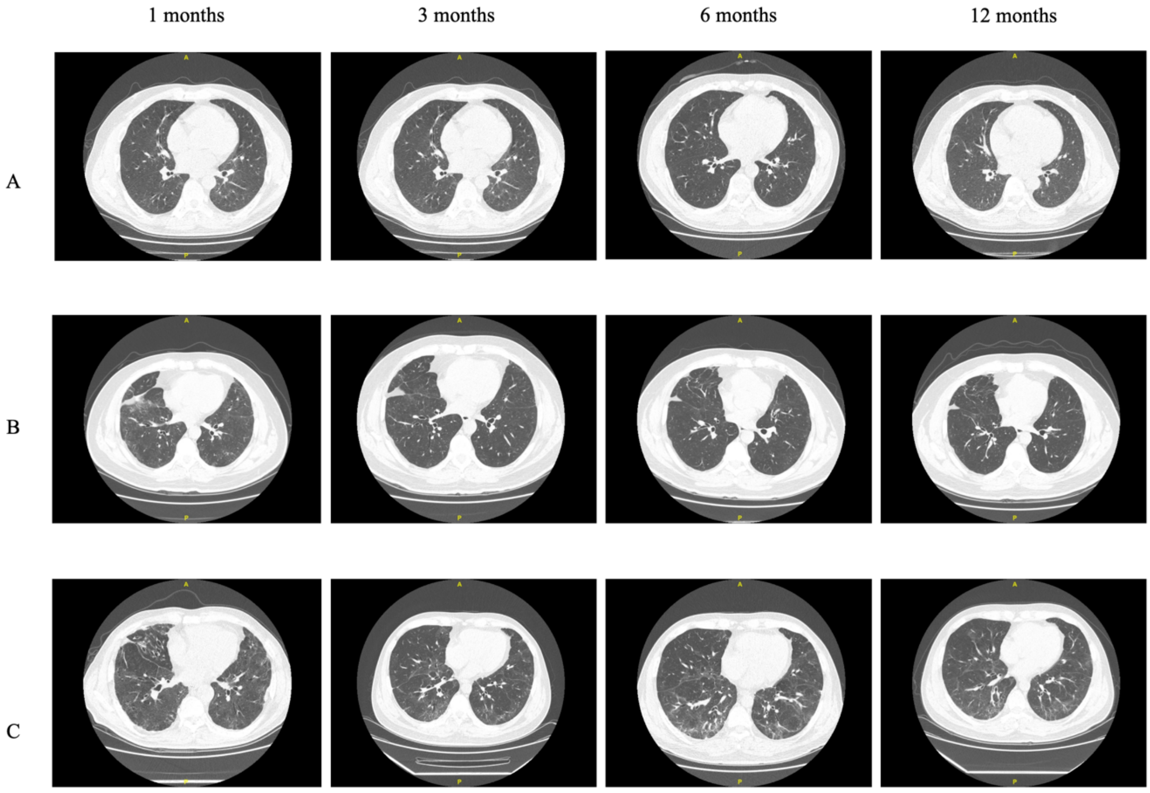

Table S1. Characteristics and outcomes of patients with H1N1 virus–induced SCAP and ARF in this cohort.

Variables	Patients (n = 92)
Gender (male, %)	70 (76.1)
Age (years)	51±14
Symptom to ICU admission (days)	8 (6, 11)
Smoking (n, %)	31 (33.7)
Acute lung injury score	3.25 (2.67, 3.75)
APACHE II	12 (9, 18)
SOFA	6 (4, 9)
Underlying disease	
Hypertension (n, %)	32 (34.8)
Coronary heart disease (n, %)	12 (13.0)
Diabetes mellitus (n, %)	16 (17.4)
Chronic kidney disease (n, %)	4 (4.3)
Chronic liver disease (n, %)	5 (5.4)
Maternal (n, %)	9 (10.8)
BMI > 30 kg/m^2 (n, %)	4 (4.3)
Immunosuppression (n, %)	3 (3.3)
Complications	
Septic shock (n, %)	17 (18.5)
Acute kidney injury (n, %)	6 (6.5)
Heart failure (n, %)	2 (2.2)
Organ support	
Noninvasive positive pressure ventilation (n, %)	47 (51.1)
Invasive mechanical ventilation (n, %)	70 (76.1)
Highest tidal volume (mL/kg)	8.5 (6.3, 10.2)
Highest minute volume (L/min)	15.6 (14.8, 19.1)
Highest respiratory rate (breaths/min)	40 (33, 44)
Highest FiO_2	1.0 (0.8, 1.0)
Highest PEEP (cmH_2O)	15 (14, 17)
Compliance (ml/cmH_2O)	25 (19, 37)
Ventilation duration (h)	457 (253, 744)
Extracorporeal membrane oxygenation (n, %)	38 (41.3)
Prone position (n, %)	52 (56.5)
Continuous renal replacement therapy (n, %)	28 (30.4)
ICU mortality (n, %)	32 (34.8)
ICU duration (days)	16 (10, 27)

Abbreviations: ARF, acute respiratory failure; APACHE, acute physiology and chronic health evaluation; BMI, body mass index; ICU, intensive care unit; PEEP, positive end expiratory pressure; SCAP, severe community-acquired pneumonia; and SOFA, sequential organ failure assessment.

Table S2. Correlation of DLCOcSB of predict values in pulmonary function with HRCT manifestations and SF-36 instrument during 12 months of follow-up after ICU discharge.

		DLCOcSB of predict values	
		Correlation coefficient	*P*
Reticulation or interlobular septal thickening	1-month	−0.475	0.011
	3-month	−0.471	0.004
	6-month	−0.442	0.027
	12-month	−0.504	0.020
SF-36			
Physical functioning	1-month	0.419	0.024
	3-month	−0.028	0.883
	6-month	0.066	0.764
	12-month	0.205	0.138
Role–physical	1-month	0.464	0.011
	3-month	0.683	<0.001
	6-month	0.515	0.012
	12-month	0.341	0.096
Bodily pain	1-month	0.367	0.050
	3-month	0.622	<0.001
	6-month	0.606	0.002
	12-month	0.431	0.032
General health	1-month	0.340	0.071
	3-month	0.228	0.226
	6-month	0.455	0.002
	12-month	0.491	0.032
Vitality	1-month	0.179	0.352
	3-month	0.168	0.113
	6-month	0.180	0.411
	12-month	0.217	0.298
Social functioning	1-month	0.373	0.046
	3-month	0.211	0.263
	6-month	0.271	0.210
	12-month	0.389	0.055
Role–emotional	1-month	0.375	0.045
	3-month	0.507	0.004
	6-month	0.434	0.039
	12-month	0.105	0.618
Mental health	1-month	0.226	0.238
	3-month	0.384	0.036
	6-month	−0.117	0.593
	12-month	0.081	0.700
Reported health transition	1-month	0.620	<0.001
	3-month	0.361	0.050

	6-month	0.254	0.242
	12-month	0.134	0.523
6MWT	1-month	0.174	0.357
	3-month	0.522	0.003
	6-month	0.184	0.413
	12-month	0.259	0.212

Abbreviations: ICU, intensive care unit; DLCOcSB, diffusion capacity of the lung for carbon monoxide at single breath; HRCT, high-resolution computed tomography; and 6MWT, 6-minute walk test.

Table S3. Correlation of HRCT manifestations with fibrogenic cytokines during 12 months of follow-up after ICU discharge.

		Reticulation or interlobular septal thickening	
		Correlation coefficient	*P*
KL-6	1-month	0.393	0.018
	3-month	0.340	0.032
	6-month	0.019	0.921
	12-month	0.053	0.782
Type III procollagen	1-month	0.282	0.100
	3-month	0.273	0.093
	6-month	0.416	0.028
	12-month	0.375	0.045
Type IV collagen	1-month	0.268	0.115
	3-month	0.276	0.085
	6-month	0.385	0.039
	12-month	0.363	0.049
Laminin	1-month	0.289	0.114
	3-month	0.026	0.882
	6-month	0.640	<0.001
	12-month	0.549	0.003
Hyaluronic acid	1-month	0.010	0.960
	3-month	−0.130	0.472
	6-month	0.530	0.005
	12-month	0.458	0.021

Abbreviations: ICU, intensive care unit; HRCT, high-resolution computed tomography; and KL-6, Krebs Von den Lungen-6.

Table S4. Collinearity diagnostics of reticulation or interlobular septal thickening and other variables at the 12-month follow-up after ICU discharge.

	P	Collinearity diagnostics	
		Tolerance	VIF
Acute lung injury score	0.242	0.902	1.108
APACHE II	0.807	0.259	3.864
Role–physical	0.892	0.721	1.387
General health	0.660	0.483	2.070
Highest tidal volume	0.228	0.277	3.616
DLCOcSB of predict	0.569	0.134	7.443

Abbreviations: APACHE, acute physiology and chronic health evaluation; ICU, intensive care unit; DLCOcSB, diffusion capacity of the lung for carbon monoxide at single breath; and VIF, variance inflation factor.
